# Gene Expression Signatures of Extracellular Matrix and Growth Factors during Embryonic Stem Cell Differentiation

**DOI:** 10.1371/journal.pone.0042580

**Published:** 2012-10-15

**Authors:** Rekha Nair, Alyssa V. Ngangan, Melissa L. Kemp, Todd C. McDevitt

**Affiliations:** 1 The Wallace H. Coulter Department of Biomedical Engineering, Georgia Institute of Technology and Emory University, Atlanta, Georgia, United States of America; 2 The Parker H. Petit Institute for Bioengineering and Bioscience, Georgia Institute of Technology, Atlanta, Georgia, United States of America; University of Pittsburgh, United States of America

## Abstract

Pluripotent stem cells are uniquely capable of differentiating into somatic cell derivatives of all three germ lineages, therefore holding tremendous promise for developmental biology studies and regenerative medicine therapies. Although temporal patterns of phenotypic gene expression have been relatively well characterized during the course of differentiation, coincident patterns of endogenous extracellular matrix (ECM) and growth factor expression that accompany pluripotent stem cell differentiation remain much less well-defined. Thus, the objective of this study was to examine the global dynamic profiles of ECM and growth factor genes associated with early stages of pluripotent mouse embryonic stem cell (ESC) differentiation. Gene expression analysis of ECM and growth factors by ESCs differentiating as embryoid bodies for up to 14 days was assessed using PCR arrays (172 unique genes total), and the results were examined using a variety of data mining methods. As expected, decreases in the expression of genes regulating pluripotent stem cell fate preceded subsequent increases in morphogen expression associated with differentiation. Pathway analysis generated solely from ECM and growth factor gene expression highlighted morphogenic cell processes within the embryoid bodies, such as cell growth, migration, and intercellular signaling, that are required for primitive tissue and organ developmental events. In addition, systems analysis of ECM and growth factor gene expression alone identified intracellular molecules and signaling pathways involved in the progression of pluripotent stem cell differentiation that were not contained within the array data set. Overall, these studies represent a novel framework to dissect the complex, dynamic nature of the extracellular biochemical milieu of stem cell microenvironments that regulate pluripotent cell fate decisions and morphogenesis.

## Introduction

For three decades, embryonic stem cells (ESCs) have been used as a model of mammalian developmental morphogenesis in order to define and characterize mechanisms of self-renewal and differentiation of pluripotent cells [1], [2], [3]. The pluripotent differentiation potential of ESCs and derivation of human ESCs have also motivated the pursuit of regenerative ESC-based therapies to restore endogenous cells to tissues afflicted by traumatic injuries or chronic disease [4], [5]. In recent years, an increasing emphasis has also been placed on the potent paracrine morphogenic effects of molecules synthesized and secreted by stem cells, including ESCs [6], [7], [8]. *In vitro*, ESCs are commonly induced to differentiate via the spontaneous assembly of cell aggregates in suspension referred to as “embryoid bodies” (EBs) [9], [10]. The temporal sequence of ESC differentiation that occurs spontaneously within EBs recapitulates several aspects of early embryogenesis, including gastrulation of the cells to yield derivatives of the three germ lineages – ecto-, endo-, and mesoderm [4], [9]. While most studies of ESC differentiation have focused on the temporal expression changes of intracellular signaling molecules and phenotypic markers that accompany differentiation, the complex patterns of extracellular molecule expression by differentiating pluripotent stem cells that can significantly influence cell phenotype(s) in an autocrine and/or juxtracrine manner remain less well defined.

Exogenous administration of ECM and growth factor molecules in different combinations and sequences has been used to direct the differentiation of stem cells *in vitro* to specific cell phenotypes by precisely controlling the biochemical composition of the cell microenvironment. This strategy, often inspired by principles of developmental biology, has been successfully employed to direct pluripotent stem cell differentiation to certain cell types [11], [12], [13], but many protocols remain inefficient and difficult to reproduce. For example, growth factors such as bone morphogenetic proteins (BMPs), fibroblast growth factors (FGFs), and vascular endothelial growth factor (VEGF), as well as adhesive ECM proteins, such as collagens, laminins and fibronectin, have been commonly applied to promote the differentiation of particular cell fates [14], [15], [16], [17]. The development of high-throughput combinatorial array technologies can facilitate more rapid, parallel screening of ESC differentiation effects by a broad contingent of ECM and growth factor molecule combinations [18], [19]. Yet despite the insights gained from studies of exogenously applied extracellular molecules, the patterns of inherent ECM and growth factor expression by ESCs as a consequence of differentiation remain less well defined. Some initial work has examined the gene expression profile of both early and late EBs using microarrays [20], [21], [22], [23], [24], while others have investigated aspects of the EB transcription factor [25] and glycomics profiles [26]. In addition, the endogenous production of specific individual matrix proteins has been found to correlate with specific cell phenotypes [27], [28], [29], suggesting that the molecules produced locally within EBs can influence ESC differentiation. Thus, globally examining the temporal profile of endogenous ECM and growth factor gene expression by ESCs during the course of EB differentiation could yield new insights into the extracellular factors regulating the different stages of pluripotent stem cell differentiation.

The objective of this study was to globally assess the dynamics of ECM and growth factor expression associated with the differentiation of ESCs within the EB microenvironment using PCR arrays for gene expression and pathway analyses. Gene expression of EB differentiation focused on ECM components, including cell adhesion molecules, matricellular proteins, integrins, and proteases, as well as growth factors, including members of the BMP, FGF, transforming growth factor β (TGFβ), and interleukin (IL) families. Gene expression profiles were contrasted using hierarchical clustering, k-means clustering, and statistical mapping to identify different global patterns of expression, as well as shared profiles of independent molecules; the combination of these approaches enabled the identification of groups of molecules expressing either coincident or divergent expression patterns. Subsequent pathway analyses highlighted key signaling pathways capable of acting on transcription factors regulating ESC phenotype at different stages of differentiation that were reconstructed solely from ECM and growth factor gene expression data. Characterizing the dynamic relationships between ECM/growth factor expression and EB differentiation using the quantitative analytical framework described provides new insights into the composition of the extracellular microenvironment regulating pluripotent stem cell biology and associated with early morphogenic differentiation events.

## Methods

### Embryoid Body Culture

Mouse embryonic stem cells (ESCs; D3 cell line obtained from ATCC) were initially expanded on a feeder layer of mouse embryonic fibroblasts and subsequently cultured feeder-free on 0.1% gelatin-coated polystyrene cell culture dishes (Corning) with Dulbecco's modified eagle medium (Mediatech), supplemented with 15% fetal bovine serum (HyClone), 2 mM L-glutamine (Mediatech), 1× MEM non-essential amino acid solution (Mediatech), antibiotic/antimycotics (Mediatech), 0.1 mM β-mercaptoethanol (MP Biomedicals, LLC), and 10^3^ U/mL leukemia inhibitory factor (LIF) (ESGRO). ES cells were passaged every two to three days before reaching ∼70% confluence. To initiate EB culture, adherent ESCs were detached from the gelatin-coated dishes using 0.05% Trypsin/0.53 mM EDTA (Mediatech) and 100 mm bacteriological grade polystyrene Petri dishes (Corning) were inoculated with 10 mL of a suspension of ESCs (4×10^5^ cells/mL) in differentiation media (ESC media without LIF). EB suspension cultures were maintained on rotary orbital shakers (Barnstead Lab-Line, Model 2314) at 40 rpm at 37°C in 5% CO_2_ for the entire duration of suspension culture. Previous work from our lab has demonstrated that rotary orbital suspension culture methods result in greater yields of homogeneous populations of EBs [30]. Thus for this study, EBs were cultured in rotary suspension for up to 14 days.

### Microscopy and histological analysis

EB morphology was monitored daily by phase microscopy for up to 14 days of differentiation using a TE2000 microscope (Nikon) and a Spot Flex camera (Diagnostic Instruments, Inc.). For histological analysis, EBs collected at different stages of differentiation (4, 7, 10, or 14 days) were fixed with 10% formalin for 30 minutes and embedded in Histogel® (Richard-Allan Scientific). Histogel®-embedded samples were dehydrated through a series of alcohol and xylene rinses prior to paraffin embedding. Sections of EB samples (5 µm each) were obtained using a Microm HM 355S rotary microtome and stained with hematoxylin and eosin (H&E) using a Leica AutoStainer XL. Stained slides were coverslipped using low viscosity mounting medium (Cytoseal™ 60) and imaged on a Nikon 80i microscope equipped with a SPOT Flex Camera (Diagnostic Instruments).

### Quantitative reverse-transcription polymerase chain reaction (qRT-PCR)

RNA was extracted from undifferentiated ESCs and EBs at days 4, 7, 10, and 14 of differentiation (n≥3 for each sample) using the RNeasy Mini Kit (Qiagen). Complimentary DNA was reverse transcribed from 1 µg of total RNA using the iScript cDNA synthesis kit (Bio-Rad), and real-time RT-PCR was performed using SYBR Green technology with a MyiQ cycler (Bio-Rad). Beacon Designer software was used to design forward and reverse primers for pluripotency and differentiation markers as well as for the housekeeping gene glyceraldehyde-3-phosphate dehydrogenase (*Gapdh*), which were each independently validated with appropriate positive cell controls. Relative levels of pluripotent gene expression were calculated compared to undifferentiated ESC samples and normalized to *Gapdh* using the ΔΔC_t_ method [31], whereas gene concentrations for differentiated markers were calculated based upon standard curves and normalized to *Gapdh* expression levels.

For SuperArray RT^2^ Profiler™ PCR array analysis, cDNA synthesis was performed using the SuperArray RT^2^ First Strand kit (SABiosciences). Genomic DNA was eliminated by mixing 0.5 µg RNA with 5× gDNA Elimination Buffer and RNase-free water before being incubating at 42°C for 5 minutes. The RT cocktail (5× RT Buffer 3, Primer & External Control Mix, RT Enzyme Mix 3, and RNase-free water) was prepared and added to the elimination buffer mixture. Each cDNA sample was synthesized in an iCycler Thermal Cycler (Bio-Rad) (15 minutes at 42°C, 5 minutes at 95°C) and diluted with RNase-free water after synthesis was complete. RT-PCR was performed by first preparing the experimental cocktail (2× SuperArray RT^2^ qPCR Master Mix, first strand cDNA synthesis reaction, and RNase-free water) and then equally distributing the cocktail (25 µL) into all of the individual wells of the PCR 96-well array (Mouse Extracellular Matrix and Adhesion Molecules array or Mouse Growth Factors array). Only one gene, secreted phosphoprotein 1 (*Spp1*), overlapped between both arrays besides the housekeeping genes and internal controls. Each array was tightly sealed with optical thin-wall 8-cap strips and amplified in a MyiQ cycler (Bio-Rad) with a two-step cycling program (1 cycle, 10 minutes, 95°C; 40 cycles, 15 seconds, 95°C; 40 cycles, 1 minute, 60°C). Fold changes in gene expression were analyzed using the ΔΔC_t_ method of quantitation, whereby samples of EBs from different time points (days 4, 7, 10, and 14) were compared relative to undifferentiated ESC values after individual sample values were normalized to internal *Gapdh* levels.

### Gene clustering analysis: hierarchical clustering and k-means

Gene expression differences by differentiating EBs were calculated as fold change increases or decreases at the different time points examined compared to ESCs, using *Gapdh* as the normalization gene as described above. Initially, the results of the independent ECM and Growth Factor PCR arrays were separately analyzed by Genesis (Release 1.7.5) array analysis software. Two-dimensional hierarchical clustering of the log2-transformed data sets was performed across the different genes and time points using Euclidean distance and average linkage clustering. The clustering results were represented visually by a heat map dendrogram, with green indicating decreased expression and red indicating increased expression relative to undifferentiated ESCs. The relative color intensity values corresponding to the magnitude of fold change (either an increase or a decrease) were set between −7.0 and 7.0 to provide a distinct color range for all log-transformed magnitudes.

Prior to all further analysis, the ECM and Growth Factor array data sets were combined and examined together. The average fold-change values of ESCs and EBs for each gene from the entire time course were analyzed using k-means clustering analysis in Genesis software (version 1.7.5). In order to determine the optimal number of k-means clusters that sufficiently captured the different distinct profiles of the entire data set, the cluster number was varied between 4 and 20 and evaluated for a maximum of 300 iterations. Analysis with fewer clusters (4–8) did not distinguish different patterns of expression as clearly, whereas larger numbers of clusters (>12) yielded some independent groups with as a few as 1–2 genes; therefore, subsequent k-means analysis was performed using 12 clusters.

### Statistics

Significance testing was conducted using SYSTAT (Version 12) software. For individual genes, expression fold change comparisons across time points were evaluated using a one-way analysis of variance (ANOVA) with subsequent post-hoc Tukey analysis to determine significance (*p*<0.05). Significant differences in expression fold-change between consecutive time points were depicted with a branch schematic for all genes from the array, with increasing or decreasing slopes representing positive or negative fold differences, respectively, while non-significant differences were represented as horizontal lines.

### Ingenuity Pathway Analysis (IPA)

Pathway analysis of genes that exhibited significant changes over time was performed using Ingenuity Pathway Analysis (Version 7.5, Ingenuity® Systems) to examine the biological functions and signaling pathways that were implicated in EB development. For each time point (days 4, 7, 10, and 14), fold-changes (−46.25 to −1 and 1 to +513.58) were filtered in IPA using a minimum 2-fold change threshold; from these genes, a list of “focus” genes was generated that contained molecules present in IPA's knowledge database (78% of eligible molecules). The top biological functions and networks for each time point were assessed based on the resulting eligible genes from the array (“focus genes”) and IPA's database containing gene associations (via physical or biological interactions). Biological functions were tested for significance (*p*<0.01) using the Benjamini-Hochberg (B–H) multiple testing correction method to account for false-positives and were then ranked in decreasing order of significance (−log (B–H p-value)). Based on published literature reports, IPA generated networks that included interactions between the focused sets of array molecules as well as other molecules present in the IPA database. Each of the networks generated by IPA, which included up to 25 focus genes, was assigned a relative score reflecting the probability that any given gene in a particular network was present by chance; higher scores indicate a lower likelihood (i.e. if probability of random gene placement = 10^−x^, then score = x).

## Results

### EB differentiation

**Figure 1:**
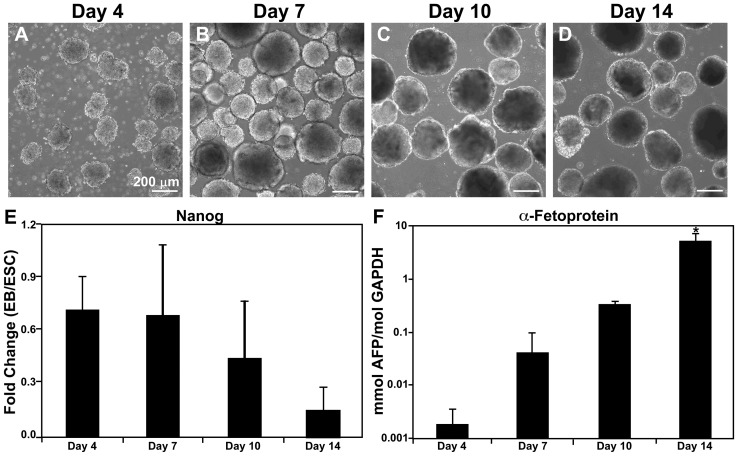
Embryoid body morphology and differentiation. (A–D) EBs cultured using rotary orbital culture maintain morphological homogeneity and increase in size over the course of differentiation. (E) Gene expression of the pluripotent marker *Nanog*, relative to ESC levels, decreases as EB differentiation proceeds. (F) Conversely, gene expression of α*-fetoprotein* (*Afp*), a marker of endoderm differentiation, increases significantly by day 14. * ANOVA: p<0.05 compared to all other time points.

The time course of EB differentiation was examined by morphology and phenotypic markers prior to performing semi-global gene expression analysis. Using rotary orbital suspension culture, the formation of EBs and maintenance of the EB population remained relatively uniform over the time course examined (Figure 1A–D), similar to previously published reports [30], [32]. Over time, the average fold change of *Nanog*, a pluripotent transcription factor (Figure 1E), decreased from 0.701±0.18 at day 4 to 0.136±0.06 by day 14 of differentiation (compared to ESCs), along with a similar decrease in *Oct-4*, another pluripotent marker (Figure S1). Conversely, the endoderm differentiation marker α-fetoprotein (*Afp*) was expressed at significantly increased levels by day 14 compared to all other time points examined (p = 0.001; Figure 1F). In addition, several genes indicative of ectoderm (*Nestin*) and mesoderm differentiation (*Gata4*, *Nkx2.5*, myocyte enhancer factor-2c, alpha myosin heavy chain, myosin light chain-2 ventricle) increased over time (data not shown), thus further confirming the expected time course of differentiation. The coincident decrease in pluripotency and increase in germ lineage marker expression, as well as the EB morphological changes that occurred over 14 days of suspension culture were consistent with previous studies from our laboratory [30], [33], [34] that reflect progressive differentiation of EB populations.

### Gene clustering based on expression profiles

**Figure 2:**
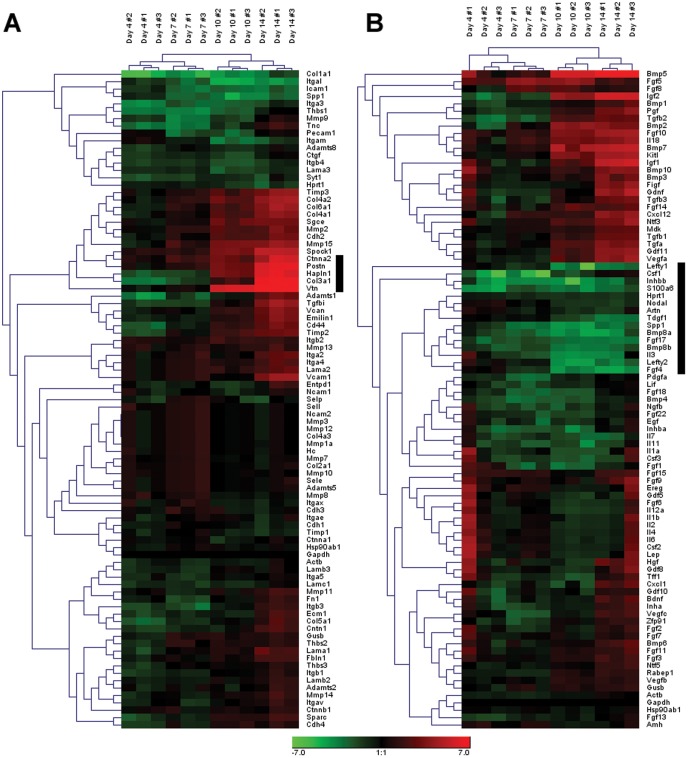
Hierarchical clustering of ECM and growth factor genes. Gene expression fold changes compared to undifferentiated ESCs were log2-transformed and analyzed using hierarchical clustering on Genesis software. Of the 84 extracellular matrix genes analyzed (A), approximately 34% of the genes demonstrated either an overall decrease (green) or increase (red) in gene expression relative to undifferentiated ESCs, whereas approximately 60% of the 84 growth factor genes (B) resulted in overall expression changes relative to ESCs. Black bars highlight noticeable gene clusters in each array set.

In order to visualize the global gene expression patterns exhibited by EBs and to identify any subsets of molecules undergoing coincident expression changes over time, two-dimensional hierarchical clustering was performed independently for the Extracellular Matrix and Adhesion Molecules array (referred to subsequently as the ECM array; Figure 2A) and the Growth Factors array (Figure 2B). In both the ECM and Growth Factor arrays, independent replicate EB samples examined at each of the individual time points clustered most closely together, indicating the reproducibility of the experimental samples. In addition, the earlier time points examined (4 and 7 days) clustered separately from the later time points (10 and 14 days), reflecting the distinct shifts in gene expression expected to occur over the course of differentiation. For each array, increasing (red) and decreasing (green) patterns of gene expression over time were evident, while a number of genes also appeared relatively unchanged compared to the original undifferentiated ESCs over the same period of differentiation time. In the ECM array, ∼18% of the molecules (16 genes) clustered together appeared to decrease considerably over time relative to ESC levels, while ∼16% (14 molecules) consistently increased over the course of differentiation. The remaining 66% (59 molecules) exhibited nominal changes roughly equivalent to baseline values of undifferentiated ESCs. In contrast to the ECM array, there were a greater number of genes from the Growth Factor array that clustered together due to their increasing (∼29%; 26 molecules) or decreasing (∼31%; 28 molecules) expression patterns. Within these relatively broad classifications, visibly distinct clusters emerged from both arrays, including five sharply increasing molecules in the ECM array that exhibited 46- to 513-fold increases compared to ESCs by day 14 (indicated by the black bar, Figure 2A). In contrast, 15 molecules in the Growth Factor array clustered together that exhibited as much as a 25-fold decrease compared to ESCs (black bar, Figure 2B). Overall, a larger percentage of genes in the Growth Factor array (∼60%) exhibited either increasing or decreasing fold changes than genes contained within the ECM array (34%). Identifying such groups of genes in any data set may lead to the classification of subgroups that are similarly regulated over the given condition, but changes in the magnitude of gene expression alone are not necessarily sufficient to accurately yield insights into the key regulators of the system.

**Figure 3:**
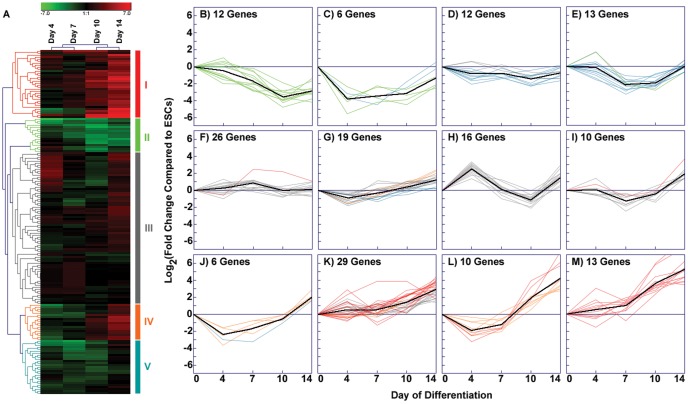
K-means clustering of combined array sets. (A) The hierarchical cluster produced by merging data from both the ECM and growth factor arrays exhibits several identifiable clusters, indicated by groups I–V. (B–M) K-means clustering of the combined data set highlights the subtle temporal changes in gene expression while clustering genes that follow similar patterns of expression over time. In the k-means graphs, the x-axis at zero represents the undifferentiated ESC baseline. The centroid of each cluster is indicated by a black line (Figure 3B–M), while colored lines in each panel correspond to genes in the color-coded groups (I–V) established by hierarchical clustering (A).

In addition to examining the ECM and Growth Factor arrays individually, a complete heat map of the entire set of experimental data from both arrays was generated from the average fold-change values of each gene at the individual time points examined (Figure 3A). Hierarchical clustering identified five primary groups of genes (labeled I–V) based on the relative gene expression patterns over time (Table S1). The genes in groups I and IV increased over the course of differentiation, while genes in groups II and V decreased, and those in group III remained relatively unchanged compared to ESCs. In order to more clearly define the temporal expression patterns embedded within the data, k-means analysis of the log2-transformed fold changes was performed (Figure 3B–M). As described in the methods, k-means analysis with a k-value of 12 captured an array of different expression patterns with a range of 6–29 genes per cluster (Table S2).

The k-means analysis highlighted the dynamic nature of gene expression values associated with temporal changes of EBs. Few genes exhibited continually increasing or decreasing profiles and only one centroid (black line) consistently increased between each of the different time points examined (Figure 3M). In the four k-means clusters where the centroid tended to decrease throughout differentiation (Figure 3B–E; 43 genes, ∼25% of the total number), genes consisted primarily of hierarchical cluster groups II and V. This demonstrated the ability of k-means to distinguish similarly expressed patterns of genes associated by the overall hierarchical clustering algorithm. In addition, genes that appeared to change nominally over time by hierarchical clustering analysis (group III) were spread among four different k-means plots (Figure 3F–I; 71 genes, ∼41%), each containing centroids that exhibited differing profiles between the examined time points. Similarly, genes that clustered in groups I and IV were primarily distributed amongst four k-means plots (Figure 3J–M; 58 genes, ∼34%) that almost exclusively contained genes exhibiting larger increases at later stages (post-day 7) of differentiation. Overall, k-means cluster analysis further refined patterns of temporal gene expression changes initially generated from hierarchical clustered data and thereby enabled the identification of several additional groups of ECM molecules and growth factors sharing similar temporal expression profiles during the course of differentiation.

### Parallel analysis of variance (ANOVA) significance testing

**Figure 4:**
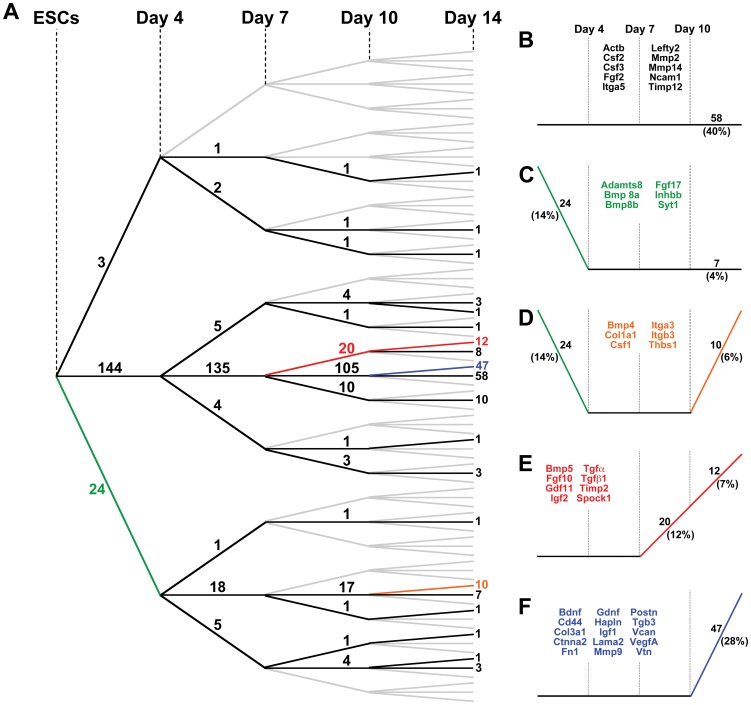
Parallel ANOVA analysis. (A) ANOVAs were performed across each time point for individual genes from both ECM and growth factor arrays. Results are depicted using a tree schematic, where the slope of the line between each time point indicates a significant increase (positive slope), significant decrease (negative slope), or no significance (zero slope). The number of genes in individual categories is shown on each line. A majority of the genes analyzed do not change significantly by 14 days of EB culture; however, a large number of genes significantly increase in expression between days 10 and 14. (B–F) Each graph highlights individual profiles of statistical changes in gene expression, including the number of genes encompassed in the respective profile and a sample of specific genes.

Although hierarchical and k-means clustering analysis enabled the identification of subgroups of genes that were similarly expressed over the course of differentiation, the correlative relationships were based upon the magnitudes of fold-change relative to the starting state (ESCs), but didn't account for statistical changes occurring between each of the discrete time points examined. Parallel independent ANOVA analysis for each gene was therefore performed to evaluate significant changes in gene expression over time (Figure 4). In general, the number of genes exhibiting significant differences in expression between time points increased with the progression of development. Approximately 16% of the genes examined changed significantly between day 0 (ESCs) and day 4, 10% between days 4 and 7, ∼22% between days 7 and 10, and ∼44% between days 10 and 14. By day 4 of differentiation, ∼89% of the molecules that changed significantly exhibited decreases in gene expression compared to ESCs, whereas by day 14, ∼99% of genes that changed significantly were increased compared to day 10. These contrasting patterns suggest that decreased expression of a small subset of extracellular factors followed by increased expression of many matrix and growth factor morphogens accompany phenotypic and morphological changes of ESCs undergoing differentiation within EBs.

The results of the ANOVA analyses were depicted in the form of a statistical “tree” to distinguish the numbers of genes that were increasing, decreasing, or not changing significantly between each of the differentiation time points examined (Figure 4A). A third of the genes (58 out of 171 total) did not change significantly throughout the entire 14-day EB culture period (Figure 4B), including *Fgf2*, matrix metalloproteinase 2 (*Mmp2*), and the housekeeping genes heat shock protein 90 kDa alpha, class B member 1 (*Hsp90ab1*) and β-actin (*Actb*). In the previous analyses, hierarchical and k-means clustering results similarly indicated that expression of both *Hsp90ab1* (Figure 3A, F) and *Fgf2* (Figure 3A, I) did not vary with time. In contrast, the *Mmp2* (Figure 3A, K) and *Actb* (Figure 3A, D) profiles generated by the clustering methods suggested increasing and decreasing expression over time, respectively, indicating that conventional clustering analyses alone may not accurately convey the truly significant differences that emerge with progressive differentiation. Importantly, several distinct groups of genes that did change significantly during EB differentiation could be extracted from the statistical tree results, with each containing 4–27% of the total number of the molecules contained within the arrays (Figure 4C–F; Table S3).

Interestingly, specific families of molecules often tended to appear within the same statistical “branches”, suggesting that related molecules may be coordinately regulated during the course of EB differentiation. For example, cadherins (4/4 genes analyzed in the array), fibroblast growth factors (6/17 genes), interleukins (10/10 genes), and matrix metalloproteinases (4/12 genes) all appeared within the group of genes that did not change significantly between consecutive time points (Figure 4B), whereas members of the transforming growth factor (5/5 genes), laminin (3/6 genes), and collagen (5/8 genes) families exhibited coordinate changes, such as increasing significantly between days 10 and 14 (Figure 4E, F). The increasing diversity of molecular expression profiles that appeared over the entire 14-day EB time course of examination was presumably reflective of the biochemical complexity of the extracellular EB environment. Mapping significant changes in gene expression over time provides a global view of matrix signatures that can highlight subgroups of statistically related molecules, independent of magnitude changes, whose coordinated increase or decrease in expression may be related to the course of specific differentiation events.

### Pathway Analysis

**Figure 5:**
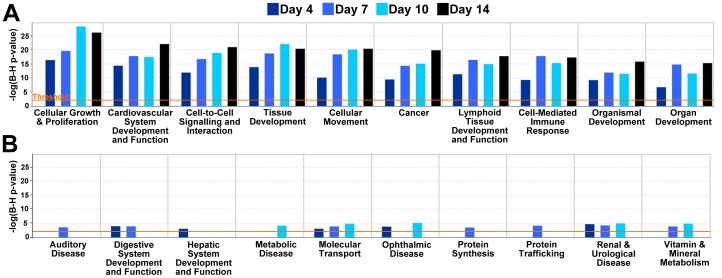
Biological functions related to global gene expression changes. Graphs of biological functions were generated using “focus” genes and the database of gene interactions present within Ingenuity Pathway Analysis software presented according to day 4 EB ranking results. (A) The ten highest-ranked biological functions that were significant at different differentiation time points consisted of processes typically related to development, including morphogenesis and proliferation. (B) In contrast, the ten lowest-ranked biological processes included disease states not typically associated with development.

Network analysis was performed using only the genes that changed significantly over the course of differentiation and exhibited more than two-fold expression changes compared to undifferentiated ESCs. Using the IPA database, biological functions reflected by these genes were generated and ranked (only the 10 highest- (A) and 10 lowest-ranked (B) functions are shown in Figure 5). Processes that contribute to critical aspects of embryonic morphogenesis consistently appeared among the top 10 functions for each time point, including “cell growth & proliferation”, “tissue development”, “cell movement”, and “cardiovascular system development & function” (Figure 5A). In contrast, the lowest ranking functions (Figure 5B) largely included disease states not conventionally related with development, such as “auditory disease”, “metabolic disease”, and “renal and urological disease”; the absence of bars in some graphs indicate instances where no array gene met the aforementioned threshold criteria. Altogether, the quantitative ranking of these functions suggested that the gene expression analysis of ECM and growth factors alone was capable of predicting the sorts of global phenotypic changes that are known to accompany ESC morphogenesis within EBs.

**Figure 6:**
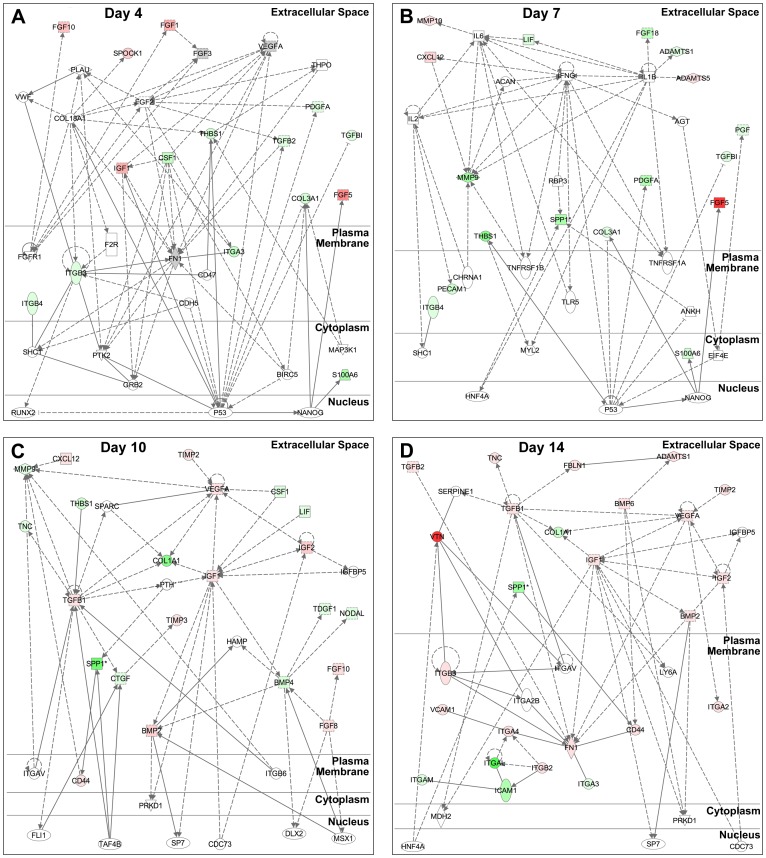
Gene networks identified during early EB differentiation. Network analysis using Ingenuity Pathway Analysis software was performed on genes that changed significantly over the course of differentiation and that exhibited at least a two-fold expression change compared to ESCs. The shift in the top networks between (A) day 4, (B) day 7, (C) day 10, and (D) day 14 demonstrates a progression from a more undifferentiated state (A) towards more differentiated ones (B–D). Relationships are divided into direct (solid line) and indirect (dashed line, - - - -) interactions, with arrow heads exhibiting the affected gene, as well as several “hub” genes. Varying intensities of green denote negative fold changes, while shades of red indicate positive fold changes. Grey genes represent genes with fold changes less than 2 that are related to other genes in the network, while genes with no color were not contained in the array data set.

The networks generated from the gene expression data highlighted the transition of the differentiating ESCs away from a pluripotent state (the top network for each time point is shown in Figure 6). Across the networks, several “hub” genes exhibited at least six network connections, including array genes fibronectin (*Fn1*; day 4), *Mmp9* (day 7), *Vegfa* (day 10), and insulin-like growth factor 1 (*Igf1*; day 14). At day 4 (Figure 6A), the top network was generated with 15 genes from the array (probability of random gene placement = 10^−23^; i.e. score = 23). Many of the molecules included in the top day 4 network were related to the *p53* transcription factor that acts to suppress the pluripotent marker *Nanog* and thereby induce differentiation [35]. The top network at day 7 (Figure 6B) contained 17 of the array genes (score = 24) as well as several extracellular factors related to *p53*; however, several growth factors that act on *p53* in the day 4 network were not present at day 7, indicating an emergence of different roles for growth factors by day 7. Along with the greater number of genes significantly increasing over time, the number of genes included in the networks at later time points also grew such that the top networks for days 10 (Figure 6C) and 14 (Figure 6D) contained 22 (score = 35) and 25 (score = 39) array genes, respectively. Interestingly, compared to earlier time points, the network at day 10 included several more nuclear factors, such as friend leukemia integration 1 (*Fli1*), TATA box binding protein-associated factor 4B (*Taf4b*), and cell division cycle 73 (*Cdc73*), linked to extracellular factors, but their connectivity was low (2–3 connections) compared to the number of connections formed to *p53* at days 4 and 7. This increase in the number of nuclear factors present in the day 10 network is suggestive of fewer commonly shared nuclear targets by the population of differentiating cells, which is consistent with the onset of diverging cell phenotypes within EBs to different germ lineages. Overall, the physical and biological network connections generated using statistically significant array data highlight the ability of ECM and growth factor expression patterns alone to elucidate global trends in cell growth and differentiation as a function of time.

## Discussion

The use of a multivariate approach to investigate extracellular matrix and growth factor expression profiles of stem cells highlighted dynamic changes among genes that occur coincidentally with differentiation. At each time point evaluated, clustering analyses revealed that subsets of genes were differentially expressed compared to ESCs. Furthermore, temporal expression changes of clustered genes demonstrated dynamic and often divergent patterns of ECM and growth factor expression during EB differentiation. Thus, expression analyses derived solely from extracellular molecules can provide insight into morphogenic processes occurring during ESC differentiation within EBs.

As ESCs differentiate within the 3D EB environment, extracellular gene expression patterns change dynamically to accompany the cell phenotype and morphogenic changes occurring. Differentiation of ESCs within EBs is usually an increasingly complex process due to the heterogeneous differentiation of the cells to divergent phenotypes. In contrast to the traditional approach of focusing on the gene expression changes of one molecule at a time, gene array analysis provides a means to analyze a large number of molecules simultaneously while monitoring subtle changes in expression to collectively analyze the system. This method of analysis may provide insight into genes that display interesting dynamic changes alongside a particular subset of genes, but that may not have been identified initially as a gene of interest for single gene PCR [27]. Importantly, ontological assessment of the extracellular genes (ECM and growth factors) included in the arrays provided links to intracellular genes, such as receptors and transcription factors, known to regulate the types of morphogenic changes that occur within the EB microenvironment.

Interestingly, the gene expression analysis of extracellular factors alone was capable of identifying established morphogenic processes known to within differentiating EBs. Clustering analysis revealed a subset of genes that are composed primarily of growth factors, which decrease sharply between days 0 and 4 prior to gradual increases at days 7 and 10. The day 4 EB network (Figure 6A) was comprised largely of growth factors (and not ECM molecules) that appear to primarily act upon the transcription factor *p53*, which in turn decreases the pluripotent marker *Nanog*. Together, these results suggest that changes in the growth factor gene expression profile of early stage-EBs accompany their exit from the core transcriptional feedback loop that regulates pluripotency. The ECM molecules present in the top day 7 EB network (Figure 6B) are largely proteases, which may serve to remodel the initial matrix present in the EBs and allow for the subsequent increase in molecules necessary to support the differentiation to different cell phenotypes. By day 10 (Figure 6C), both ECM molecules and growth factors appear in the top ranked network, along with an increase in the number of connected transcription factors that mediate ESC differentiation to divergent phenotypes. Finally, the appearance of a number of different integrin species by day 14 in the top-ranked network (Figure 6D) seems to reflect global changes in cell adhesion that can occur as different phenotypes emerge and adhesive ECM molecules are expressed.

Complementary information provided from different forms of gene expression analysis yields an overall more comprehensive characterization of the temporal dynamics of EB differentiation than individual analyses alone. Some molecules appear consistently throughout the time course and change their molecular interactions with time, while others are unique to certain stages of differentiation. As an illustrative example, *Vegfa* clustered with genes such as *Igf2* and *Tgfb2* within the growth factor array (Figure 2B, Figure 3L) and was also found to be highly related to expression patterns of matrix proteins such as *Col3a1* and *Emilin1* (Figure 3L) when the two sets of array data were analyzed collectively. Furthermore, the ANOVA analysis confirmed these correlative relationships when tested for statistical significance (Figure 4F). When visualized as a dynamically changing network, however, several additional features of *Vegfa* expression in EB differentiation emerge. First, even though its individual expression was not higher than the 2-fold change threshold by day 4, *Vegfa* exhibited a high degree of connectivity to other molecules in Figure 6A. Secondly, the high degree of connectivity for *Vegfa* (Figure 6A) that emerges with other genes such as *Tgfb2* and *Col3a1* is due to regulation through common transcription factors (e.g. *p53*). Through this analysis, functional relationships based on unrelated previous studies emerge from the data that allow for new hypothesis generation and testing regarding exogenous molecules capable of affecting ESC differentiation.

Just as each individual analysis lends insight when examining specific molecules, the range of analyses presented is critical for extracting a more global perspective of EB morphogenesis from the array data. Researchers often rely on a single method for assessing large data sets, typically either hierarchical clustering or pathway analysis, which can potentially result in overlooking molecules important to the system. For example, performing clustering (hierarchical or k-means) alone did not result in the identification of *Fn1* as an important molecule in the examined EB system, due to the fact that it didn't exhibit large fold changes over the time course examined. Further analysis using parallel ANOVAs indicates that *Fn1* expression increases significantly by day 14 (Figure 4F), along with other key molecules such as *Ctnna2*, *Postn*, *Hapln1*, *Col3a1*, and *Vtn* that are highlighted in clustering analyses. Pathway analysis demonstrated that *Fn1* is a hub gene at both day 4 and day 14, suggesting its importance throughout the differentiation time course. In contrast, consider *Spock1*, which is highlighted in hierarchical clustering as exhibiting one of the greatest increases in expression over time compared to ESCs, along with *Ctnna2*, *Postn*, *Hapln1*, *Col3a1*, and *Vtn*. K-means clustering also indicated an association between *Spock1* and *Postn*/*Vtn* (Figure 3M, Table S2), while ANOVA verified the gene's significant increase after day 7 (Figure 4E). However, *Spock1* was not included in the top networks at later time points of EB differentiation (days 7–14), although it was present in the top network at day 4 with a single connection. Analysis displayed a relationship between *Spock1* with *Col18A1*, a hub gene not included in the arrays, but highly connected to several ECM and growth factor genes contained in the arrays (*Vegfa*, *Fgf2*, and *Itgb3*), as well as other highly connected genes (*p53* and *Fn1*). The aforementioned examples of *Fn1* and *Spock1* demonstrate that a combination of analytical tools can identify potential key regulators in the differentiation system that would have otherwise been overlooked by single forms of analysis alone.

## Conclusions

Extracellular factors, including matrix molecules and growth factors which are typically not highlighted so much as transcription factors to examine differentiation patterns, exhibited different dynamic signatures during EB differentiation that accompany changes in ESCs, such as decreased pluripotency and the onset of tissue morphogenesis. Semi-global transcriptional network analysis of extracellular factors expressed by pluripotent cells in dynamic environments highlighted the potential importance of such endogenous molecules and their utility in assessing the temporal shifts in tissue morphogenesis of the system. In order to analyze the wide spectrum of matrix molecules in this study, multiple methods were used that highlighted differences in expression patterns through clustering tools and focused on the relationships between the molecules via network analysis. Subsets of genes with diverging expression values (i.e. groups that exhibited opposing signatures over time) emerged from k-means and statistical analyses. The simultaneous increase and decrease of different sets of molecules is likely necessary for the onset of many differentiation events, and the identification of these sub-groups could be critical for further understanding the coincident cell phenotype specification. This study also demonstrates the potential caveats of examining individual molecules in a one-dimensional fashion and neglecting the global systems view that contributes considerably to the fundamental understanding of emergent biology. In contrast, the combination of clustering/statistical analyses with network mapping provides a multi-faceted approach that enables a more in-depth understanding of the dynamic nature and processes of the EB microenvironment. Overall, these insights provide a better understanding of the transcriptional changes for ECM molecules and growth factors that accompany embryonic morphogenesis and thereby enable novel routes to consider engineering the differentiation of ESCs to specific lineages within EBs.

## Supporting Information

Figure S1 **Embryoid differentiation.** (A–D) Histological examination of EB differentiation with H&E staining. (E) Gene expression of *Oct-4,* marker of pluripotency, during EB differentiation decreases at later time points of differentiation. ANOVA: # *p*<0.05 compared to ESCs (day 0), † *p*<0.05 compared to days 4 and 7.
(DOCX)

Table S1 **Genes in each cluster represented in** **Figure 3A** **.** Hierarchical clustering identified five gene groups based on relative expression over time. Clusters I and IV increased over time, clusters II and V decreased over time, and cluster III maintained relatively unchanged gne expression levels over time.
(DOCX)

Table S2 **Genes in each k-means plot represented in** **Figure 3B** **.** K-means analysis defines temporal gene expression, refining patterns of expression and separating hierarchical clusters. Clusters B–E correspond to clusters II and V; clusters F–I correspond to clusters I and IV; clusters J–M correspond to cluster III.
(DOCX)

Table S3 **Genes represented in** **Figure 4(B–F)** **.** Liste of genes separated by ANOVA analysis that do change statistically over time (4C–F) as well as those that do not change statistically over time (4B).
(DOCX)
