# Supplementary figures and images for: Gene Expression Signatures of Extracellular Matrix and Growth Factors during Embryonic Stem Cell Differentiation
Source: PLoS One. 2012 Oct 15;7(10):e42580. doi: 10.1371/journal.pone.0042580 (PMC3471908; doi:10.1371/journal.pone.0042580)

**Figure S1.**

**
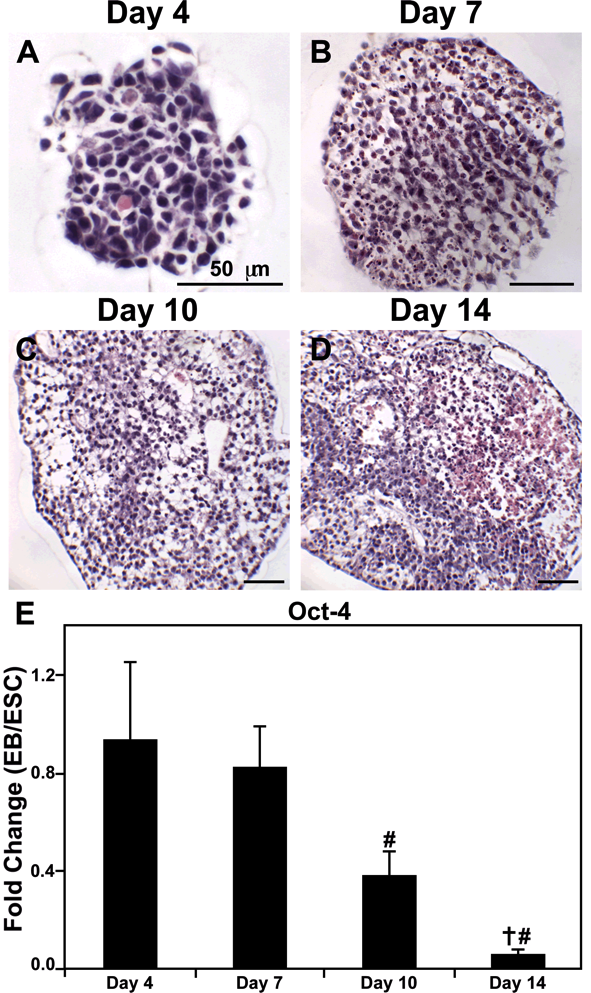
**

Supplement: Figure S1 — Embryoid differentiation. (A–D) Histological examination of EB differentiation with H&E staining. (E) Gene expression of Oct-4, marker of pluripotency, during EB differentiation decreases at later time points of differentiation. ANOVA: # p<0.05 compared to ESCs (day 0), † p<0.05 compared to days 4 and 7. (DOCX) [file pone.0042580.s001.docx]
