# Supplementary material for: Gene Expression Signatures of Extracellular Matrix and Growth Factors during Embryonic Stem Cell Differentiation
Source: PLoS One. 2012 Oct 15;7(10):e42580. doi: 10.1371/journal.pone.0042580 (PMC3471908; doi:10.1371/journal.pone.0042580)
Supplement: Table S1 — Genes in each cluster represented in Figure 3A . Hierarchical clustering identified five gene groups based on relative expression over time. Clusters I and IV increased over time, clusters II and V decreased over time, and cluster III maintained relatively unchanged gne expression levels over time. (DOCX) [file pone.0042580.s002.docx]

**Table S1. Genes in each cluster represented in Figure 3A.**

| **Cluster I** | **Cluster II** | **Cluster III** | | **Cluster IV** | **Cluster V** |
| --- | --- | --- | --- | --- | --- |
| Fgf5  Fgf8  Igf1  Bmp10  Fgf14  Ntf3  Gdnf  Figf  Bmp3  Tgfb3  Bmp2  Kitl  Mmp15  Ctnna2  Postn  Bmp7  Il18  Fgf10  Spock1  Cxcl12  Mmp2  Cdh2  Tgfa  Col4a1  Sgce  Col4a2  Col6a1  Timp3  Vcam1  Col3a1  Igf2  Hapln1  Bmp5  Vtn | Col1a1  S100a6  Itga3  Inhbb  Itgal  Bmp8b  Fgf17  Icam1  Bmp8a  Spp1  Fgf4  Lefty1  Lefty2  Tdgf1  Fgf1  Il3  Itgam | Fgf15  Fgf9  Gdf8  Hgf  Ereg  Gdf5  Il2  Il4  Il1b  Csf2  Il6  Lep  Fgf6  Il12a  Csf3  Il1a  Tff1  Fgf2  Fgf7  Gdf10  Bdnf  Cxcl1  Entpd1  Inha  Vegfc  Zfp91  Fn1  Itga2  Mdk  Itga4  Lama2  Bmp6  Fgf11  Fgf3  Itgb2  Mmp13  Rabep1  Mmp11 | Vegfb  Ctnnb1  Fbln1  Adamts2  Itgav  Lamb2  Ntf5  Mmp14  Cdh4  Thbs3  Thbs2  Gusb  Artn  Nodal  Selp  Cdh1  Timp1  Cdh3  Hc  Mmp10  Mmp7  Mmp1a  Col4a3  Mmp12  Mmp3  Ncam2  Adamts5  Col2a1  Sele  Itgax  Amh  Mmp8  Sell  Ctnna1  Ncam1  Itgae  Hsp90ab1  Gapdh | Adamts1  Col5a1  Cntn1  Ecm1  Itgb3  Tgfb2  Tgfbi  Gdf11  Vegfa  Tgfb1  Vcan  Pgf  Emilin1  Lama1  Bmp1  Sparc  Cd44  Timp2 | Csf1  Thbs1  Tnc  Pdgfa  Mmp9  Bmp4  Il11  Fgf18  Inhba  Pecam1  Adamts8  Ctgf  Syt1  Itgb4  Lama3  Il7  Hprt1  Egf  Fgf22  Ngfb  Lif  Lamc1  Fgf13  Itgb1  Lamb3  Itga5  Actb |
