# Supplementary material for: Gene Expression Signatures of Extracellular Matrix and Growth Factors during Embryonic Stem Cell Differentiation
Source: PLoS One. 2012 Oct 15;7(10):e42580. doi: 10.1371/journal.pone.0042580 (PMC3471908; doi:10.1371/journal.pone.0042580)
Supplement: Table S2 — Genes in each k-means plot represented in Figure 3B . K-means analysis defines temporal gene expression, refining patterns of expression and separating hierarchical clusters. Clusters B–E correspond to clusters II and V; clusters F–I correspond to clusters I and IV; clusters J–M correspond to cluster III. (DOCX) [file pone.0042580.s003.docx]

**Table S2. Genes in each k-means plot represented in Figure 3B.**

| **Cluster B** | **Cluster C** | **Cluster D** | **Cluster E** | **Cluster F** | | **Cluster G** |
| --- | --- | --- | --- | --- | --- | --- |
| Bmp8a  Bmp8b  Fgf17  Fgf4  Lefty1  Lefty2  Tdgf1  Icam1  Itgal  Itgam  Spp1  Il3 | Csf1  Col1a1  Itga3  Thbs1  S100a6  Inhbb | Adamts8  Timp1  Hprt1  Nodal  Lama3  Ctgf  Syt1  Il7  Itgb4  Lamc1  Actb  Artn | Egf  Fgf22  Ngfb  Fgf1  Lif  Pecam1  Csf3  Il11  Inhba  Pdgfa  Fgf18  Mmp9  Bmp4 | Fgf8  Amh  Adamts5  Ctnnb1  Cdh3  Col2a1  Col4a3  Hc  Itgae  Mmp10  Mmp12  Mmp1a  Mmp3 | Mmp7  Mmp8  Ncam2  Sele  Sell  Itgax  Hsp90ab1  Gapdh  Ctnna1  Selp  Cdh1  Ncam1  Fgf7 | Rabep1  Lamb3  Sparc  Vegfb  Cdh4  Itgb1  Lama1  Lamb2  Mmp14  Thbs3  Bmp1  Fgf13  Ntf5  Adamts2  Fbln1  Itgav  Thbs2  Gusb  Itga5 |
| **Cluster H** | **Cluster I** | **Cluster J** | **Cluster K** | | **Cluster L** | **Cluster M** |
| Gdf5  Il1b  Il2  Csf2  Ereg  Fgf15  Fgf6  Gdf10  Gdf8  Hgf  Il12a  Il1a  Il4  Il6  Lep  Tff1 | Entpd1  Vegfc  Fn1  Mmp11  Bdnf  Cxcl1  Inha  Figf  Fgf2  Zfp91 | Adamts1  Col5a1  Ecm1  Cntn1  Itgb3  Tnc | Mdk  Itga2  Itga4  Itgb2  Lama2  Mmp13  Vcam1  Bmp10  Bmp3  Bmp6  Fgf11  Fgf14  Fgf3  Fgf5  Fgf9 | Ntf3  Tgfb3  Cdh2  Mmp2  Cxcl12  Gdnf  Tgfa  Tgfb1  Col4a1  Vcan  Sgce  Timp3  Gdf11  Col6a1 | Col3a1  Emilin1  Pgf  Tgfb2  Cd44  Tgfbi  Timp2  Vegfa  Hapln1  Igf2 | Bmp7  Ctnna2  Spock1  Bmp2  Bmp5  Fgf10  Igf1  Il18  Kitl  Col4a2  Mmp15  Postn  Vtn |
