# Supplementary material for: Gene Expression Signatures of Extracellular Matrix and Growth Factors during Embryonic Stem Cell Differentiation
Source: PLoS One. 2012 Oct 15;7(10):e42580. doi: 10.1371/journal.pone.0042580 (PMC3471908; doi:10.1371/journal.pone.0042580)
Supplement: Table S3 — Genes represented in Figure 4(B–F) . Liste of genes separated by ANOVA analysis that do change statistically over time (4C–F) as well as those that do not change statistically over time (4B). (DOCX) [file pone.0042580.s004.docx]

**Table S3. List of genes represented in Figure 4B-F.**

| **Figure 4B** | | **Figure 4C** | **Figure 4D** | **Figure 4E** | **Figure 4F** | |
| --- | --- | --- | --- | --- | --- | --- |
| 57 genes | | 24 genes | 10 genes | 12 genes | 47 genes | |
| Actb | Il18 | Adamts8 | Bmp4 | Bmp5 | Adamts1 | Hapln1 |
| Adamts2 | Il1a | Bmp4 | Col1a1 | Fgf10 | Bdnf | Igf1 |
| Amh | Il1b | Bmp8a | Csf1 | Gdf11 | Bmp1 | Inha |
| Artn | Il2 | Bmp8b | Ctgf | Igf2 | Bmp3 | Itga2 |
| Bmp10 | Il3 | Col1a1 | Fgf13 | Kitl | Bmp6 | Itga4 |
| Cdh1 | Il4 | Csf1 | Itga3 | Rabep1 | Cd44 | Lama1 |
| Cdh2 | Il6 | Ctgf | Itgb3 | Spock1 | Cntn1 | Lama2 |
| Cdh3 | Il7 | Fgf13 | Itgb4 | Tgfa | Col3a1 | Lamb2 |
| Cdh4 | Inhba | Fgf17 | Lamc1 | Tgfb1 | Col4a1 | Lif |
| Csf2 | Itga5 | Fgf18 | Thbs1 | Tgfb2 | Col4a2 | Mdk |
| Csf3 | Itgae | Hprt1 |  | Timp2 | Col5a1 | Mmp11 |
| Ctnna1 | Itgav | Icam1 |  | Zfp91 | Col6a1 | Mmp9 |
| Ctnnb1 | Itgax | Inhbb |  |  | Ctnna2 | Ntf5 |
| Egf | Itgb1 | Itga3 |  |  | Cxcl1 | Pgf |
| Ereg | Lefty2 | Itgal |  |  | Cxcl12 | Postn |
| Fgf11 | Lep | Itgb3 |  |  | Ecm1 | Tgfb3 |
| Fgf2 | Mmp13 | Itgb4 |  |  | Emilin1 | Tgfbi |
| Fgf22 | Mmp14 | Lama3 |  |  | Entpd1 | Timp3 |
| Fgf6 | Mmp2 | Lamc1 |  |  | Fbln1 | Tnc |
| Fgf7 | Mmp8 | Pdgfa |  |  | Fgf3 | Vcam1 |
| Fgf9 | Ncam1 | S100a6 |  |  | Figf | Vcan |
| Gdf10 | Ngfb | Spp1 |  |  | Fn1 | Vegfa |
| Gdf5 | Ntf3 | Syt1 |  |  | Gdnf | Vegfc |
| Gdf8 | Sele | Thbs1 |  |  |  | Vtn |
| Hc | Sell |  |  |  |  |  |
| Hgf | Sparc |  |  |  |  |  |
| Hsp90ab1 | Tff1 |  |  |  |  |  |
| Il11 | Thbs3 |  |  |  |  |  |
| Il12a | Timp1 |  |  |  |  |  |
